# A rare case report of Rasmussen’s aneurysm in multidrug-resistant tuberculosis from Nepal

**DOI:** 10.1097/MS9.0000000000005612

**Published:** 2026-09-02

**Authors:** Aashish Lamichhane, Apil Pokhrel, Sadish Sharma, Bidya Adhikari

**Affiliations:** a Provincial Center for Disease Control, Pokhara, Kaski, Nepal; b Province Hospital, Beni, Myagdi, Nepal

**Keywords:** case report, hemoptysis, multidrug resistant tuberculosis, pseudoaneurysm, Rasmussen’s aneurysm

## Abstract

**Introduction and Importance:**

Rasmussen’s aneurysm is a radiological finding and an uncommon cause of hemoptysis in pulmonary tuberculosis. It results from weakening of pulmonary arterial wall due to inflammation adjacent to a tuberculous cavity.

**Case presentation:**

We report a case of 52-year-old male diagnosed with pulmonary tuberculosis, who presented with recurrent hemoptysis despite being on anti-tubercular therapy. Bacteriological examination using line probe assay revealed resistance to Rifampicin and Isoniazid, confirming multidrug resistant tuberculosis (MDR-TB). CT pulmonary angiogram (CT-PA) was performed, which demonstrated a dilated vascular channel adjacent to a cavity in the left upper lobe of the lung, suggestive of Rasmussen’s aneurysm. It was managed with trans-arterial embolization, followed by initiation of second-line regimen for MDR-TB.

**Clinical discussion:**

As Rasmussen’s aneurysm is rare, it is often overlooked when a TB case presents with massive hemoptysis. If left untreated, it could be fatal. This case highlights the utility of prompt investigation and intervention to manage the complication and prevent further deterioration.

**Conclusion:**

Rasmussen’s aneurysm is a rare cause of hemoptysis, especially in cavitary or MDR-TB. Early diagnosis with CT-PA and management with endovascular approach could be life-saving.

## Introduction

Tuberculosis (TB) continues to be one of the leading infectious causes of death worldwide^[^1,2^]^. Although the incidence of TB has declined gradually over the past few years, the burden remains stable, with nearly half of the cases occurring in the WHO South-East Asia Region (45%), followed by the African Region (24%)^[^1]. This global trend is reflected in Nepal as well, where TB remains a major public health problem, with an annual incidence rate of 229 cases per 100 000 population in Fiscal Year (FY) 2021/2022, almost equal to 238 cases per 100 000 population in FY 2020/2021^[^3,4^]^.

HIGHLIGHTS

- Rasmussen’s aneurysm is a rare but potentially fatal cause of hemoptysis in tuberculosis.
- CT pulmonary angiography is the imaging modality of choice for diagnosis.
- Trans-arterial embolization is the preferred treatment with high success rates.
- Early multidisciplinary management reduces morbidity and mortality.
- This case represents one of the few documented reports from Nepal.

TB and its control program are further complicated by the emergence of drug resistance. Resistance to rifampicin – one of the most effective first-line drugs – alone or in addition to isoniazid is called multidrug-resistant (MDR) TB, and it represents a serious therapeutic challenge. Such MDR cases need to be treated with second-line drugs that are longer, more toxic, and less effective than first-line therapy^[^1,5^]^. WHO has recognized Nepal and added it to the list of 30 high-burden MDR/RR-TB countries. This underscores the scale of the drug-resistance problem in Nepal^[^1^]^.

The primary efforts of TB control are focused on preventing transmission and drug resistance, while complications and sequelae of TB require serious clinical attention^[^2,6^]^. In this report, we present a case of MDR-TB complicated by recurrent hemoptysis due to Rasmussen’s aneurysm. This article was written in accordance with the SCARE guidelines^[^7]. The objective of this case report is to highlight Rasmussen’s aneurysm as a rare but potentially life-threatening cause of hemoptysis in MDR-TB and to emphasize the importance of early diagnosis and timely endovascular management.

## History

A 52-year-old man with a history of type 2 diabetes mellitus, chronic liver disease, and previously treated pulmonary TB (3 years ago) presented with a 3-month history of a productive cough with sputum mixed with blood. Initially, the episodes were intermittent and brief, but they became continuous over the past 20 to 22 days. He also reported decreased appetite but denied fever, night sweats, weight loss, or shortness of breath.

On assessment, the patient was hemodynamically stable. Chest radiography showed patchy opacities and fibrotic changes, predominantly in the left mid and lower lung zones, suggestive of both active TB and post-TB sequelae (Fig. 1). Sputum was sent for microscopy, culture, and GeneXpert MTB/RIF testing. Microscopy was negative for acid-fast bacilli (AFB), culture yielded normal flora, but *Mycobacterium tuberculosis* (MTB) was detected on GeneXpert without rifampicin resistance. The patient was then started on anti-tubercular therapy per the Directly Observed Treatment, Short-course (DOTS) regimen, starting on 17 May 2025. Tranexamic acid 500 mg was prescribed for 5 days.

**Figure 1.**
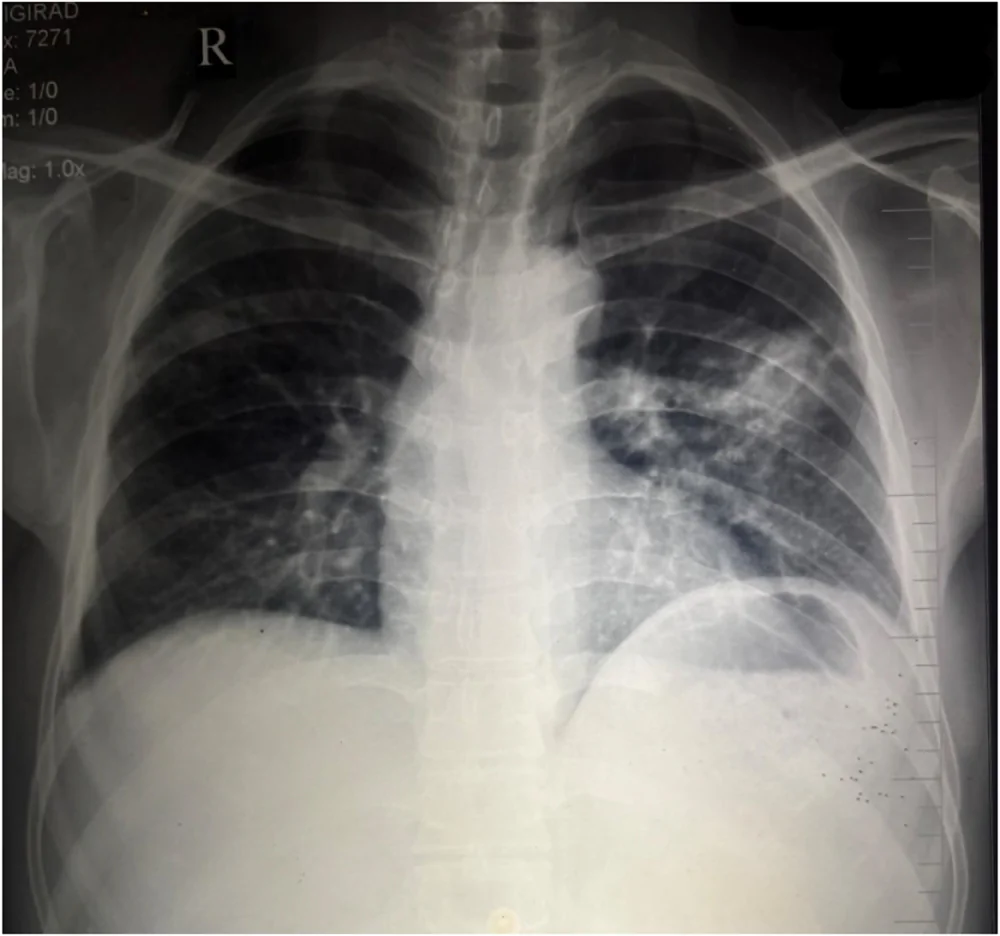
Chest X-ray showing patchy opacities over the middle and lower zones of the left lung greater than the right lung.

In the second month of follow-up, hemoptysis persisted. The bleeding was small in volume (<50 mL per episode), consistent with non-massive hemoptysis, but was recurrent. The vitals were stable and thus did not warrant emergency hospitalization or blood transfusion. Routine laboratory investigations were obtained and were mostly within expected limits; however, the patient was noted to have poorly controlled glycemia and mild anemia (Table 1). A cardiothoracic and vascular surgeon was consulted along with an interventional radiologist. Endobronchial mass, endobronchial TB, vascular causes such as bronchial artery bleeding, bronchiectasis, and aspergilloma were among the differential diagnoses considered. On 20 July 2025, bronchoscopy was performed, which was unremarkable. This ruled out endobronchial causes such as malignancy and endobronchial TB. Broncho-alveolar lavage (BAL) was collected during the procedure and sent for microbiological examination: microscopy, culture, and line probe assay (LPA). Microscopy was again negative for AFB, and culture was negative for AFB and fungi, including Aspergillus and Nocardia. However, MTB was detected in LPA, now with resistance to both isoniazid and rifampicin. This discrepancy in resistance pattern within 2 months could be attributed to heteroresistance or an initial false-negative rifampicin resistance due to low bacillary load. Moreover, LPA has a higher sensitivity for detecting resistance-associated mutations[8].

**Table 1:** Laboratory blood and urine findings.

Test	Result	Units	Reference range
Complete blood count (CBC)			
RBC	4.41	Million/µL	4.5–5.5
WBC	7290	Cells/mm^3^	4000–11 000
Platelets	213 000	Cells/mm^3^	150 000–450 000
Hemoglobin	11.2	g/dL	12–18
Renal function test (RFT)			
Serum creatinine	0.7	mg/dL	0.5–1.4
Urea	22	mg/dL	15–40
Sodium	138.3	mmol/L	135–145
Potassium	4.2	mmol/L	3.5–5.0
Random blood sugar (RBS)	224	mg/dL	<140
HbA1c	8.8	%	<5.6
Urine routine microscopy (urine RME)			
Color	Light yellow		
Albumin	Nil		
Sugar	Trace		
pH	Acidic		5.0–8.0
Specific density	1.006		1.005–1.030
Red blood cells	Nil	/High power field	0–2
Pus cells	Nil	/High power field	0–5
Epithelial cells	1-2	/High power field	0–5
Casts	Nil		

As bronchoscopy may not detect vascular causes of hemoptysis, a CT pulmonary angiogram (CT-PA) was performed. This revealed a few cavities with fibrotic walls, multiple opacities in both lung fields, and a well-defined, dilated vascular channel arising from a branch of the left pulmonary artery, projecting toward the posterior wall of the cavity in the left upper lobe – a finding consistent with Rasmussen’s aneurysm (Figs 2 and 3). On 23 July 2025, trans-arterial embolization was planned and performed with polyvinyl alcohol. The procedure was uneventful.

**Figure 2.**
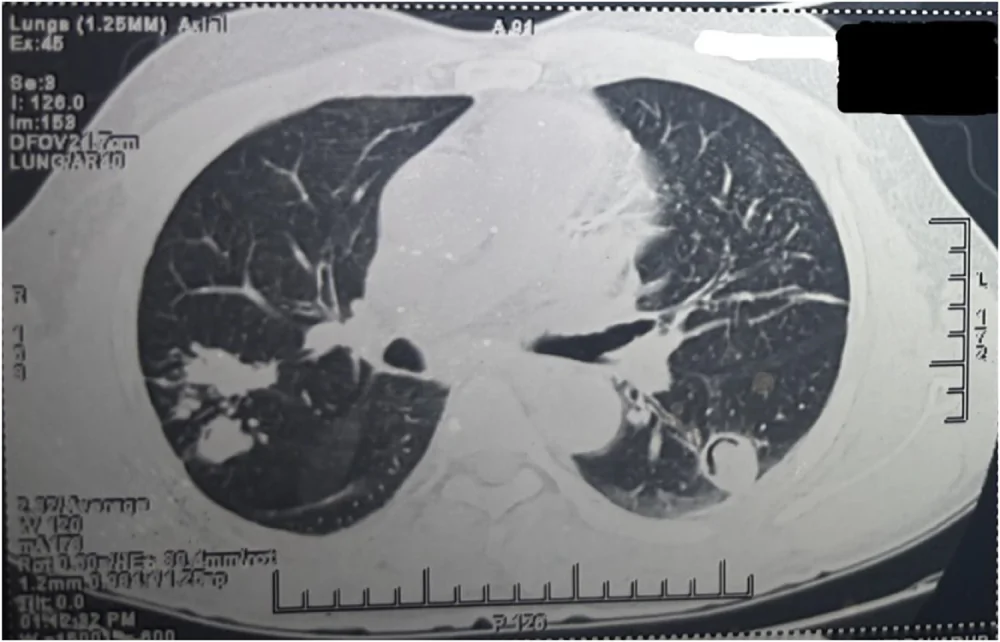
CECT chest showing multiple nodular opacities in both lungs.

**Figure 3.**
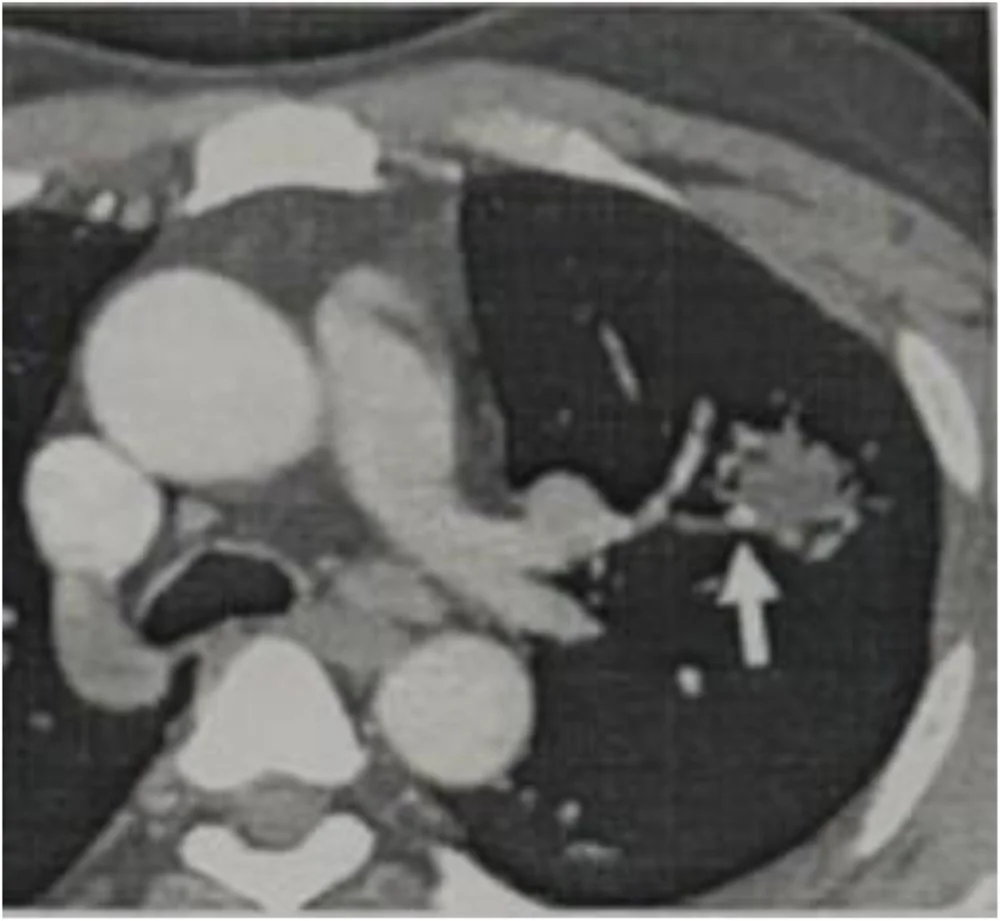
CT pulmonary angiogram (axial view) demonstrating a dilated vascular channel (white arrow) arising from a branch of the left pulmonary artery, consistent with Rasmussen’s aneurysm.

Following the LPA result, the patient was started on a 6-month BPaLM (bedaquiline, pretomanid, linezolid, and moxifloxacin) regimen for MDR-TB and was kept under regular follow-up. By the end of treatment on 20 January 2026, the patient had no new episodes of hemoptysis and was declared cured of TB. A timeline of clinical events, from diagnosis to treatment completion, is shown in Table 2.

**Table 2:** Timeline of clinical events from initial presentation to treatment completion.

Date	Events
16 May 2025	Patient presented to OPD
	Initial evaluations done
	Sputum sent for AFB, culture and GeneXpert
17 May 2025	Sputum GeneXpert positive for MTB
	DOTS started
20 July 2025	Bronchoscopy was performed
	BAL sent for culture, AFB and LPA
	MDR-TB detected
	Six-month BPaLM therapy initiated
23 July 2025	Trans-arterial embolization performed
January 20, 2026	Sixth month of follow-up
	Declared cure of TB

OPD, Outpatient Department; AFB, acid fast bacilli; MTB, *Mycobacterium tuberculosis*; DOTS, directly observed treatment short-course; BAL, broncho-alveolar lavage; LPA, line probe assay.

## Discussion

Pulmonary tuberculosis is associated with a spectrum of complications, such as hemoptysis, cavitary lesions, tuberculoma, bronchiectasis, aspergilloma, and fibrotic scarring. Hemoptysis is one of the common and clinically significant complications[6]. The source of hemoptysis in cases of TB may be the pulmonary artery, bronchial artery, intercostal artery, or any other artery that supplies the lung. Hemoptysis occurs due to various pathologies, such as bronchiectasis, cavitary erosions that extend to involve nearby vessels, or certain vascular abnormalities. Vascular lesions that are linked to TB include arteritis, thromboembolism, and aneurysm or pseudoaneurysm formation, such as Rasmussen’s aneurysm^[^9,10^]^.

Rasmussen’s aneurysm, first described by Fritz Waldemar Rasmussen in 1868, is a rare but potentially fatal complication of pulmonary TB, which has a risk of rupture leading to massive hemoptysis^[^10]. It is characterized by weakening of the arterial wall, as chronic inflammation from adjacent cavitary tuberculosis replaces the tunica adventitia and tunica media with granulation tissue. This weakened section undergoes abnormal dilatation, forming a pseudoaneurysm^[^11]. In earlier decades, during the pre-chemotherapy era, autopsy findings reported a prevalence of Rasmussen’s aneurysm of 4%, while a more recent study by Stevan *et al* (2014) found an incidence of only 0.25% among 21 532 patients with TB and hemoptysis when evaluated with CT-PA^[^12,13^]^. These findings highlight that Rasmussen’s aneurysm has long been recognized as a rare complication of pulmonary TB and should be considered especially in cases of non-resolving hemoptysis associated with pulmonary TB.

Investigation and evaluation of hemoptysis in TB are conducted in a stepwise fashion. Usually, a chest X-ray is inconclusive, revealing only parenchymal abnormalities such as opacities or cavities that are suggestive of TB, without providing much detail on the vasculature^[^14]. Bronchoscopy provides direct visualization of the airways, helps to exclude endobronchial pathology, and allows for microbiological sampling, but its success rate for detecting vascular lesions is low^[^15,16^]^. CT-PA is currently the imaging modality of choice, as it not only characterizes the underlying parenchymal and cavitary lesions but also identifies vascular abnormalities such as aneurysms and pseudoaneurysms with high sensitivity^[^17]. In our case, the combination of chest X-ray and bronchoscopy guided our initial evaluation, but early referral for CT-PA and angiographic assessment remained crucial for definitive diagnosis.

Medical management plays only a supportive role, leaving interventional and surgical procedures to treat the underlying etiology of hemoptysis. Antitubercular drugs can only control the underlying infection and cannot treat the vascular pathology. Mild to moderate hemoptysis, however, may respond to anti-tubercular drugs^[^18]. In the event of a leaking Rasmussen’s aneurysm, the foremost priority is protecting the airway and maintaining adequate oxygenation^[^19]. Once stabilized, bleeding control should be promptly attempted through trans-arterial embolization, which remains the preferred modality^[^18]. Trans-arterial embolization is considered the first-line treatment whenever feasible because of its low morbidity and mortality, and a success rate exceeding 90%^[^20]. Additionally, patients with severe hemoptysis are poor surgical candidates due to underlying compromised respiratory function, further underscoring the role of embolization as a preferred modality^[^21]. Surgical resection, such as lobectomy or segmentectomy, is reserved particularly for those with localized disease, recurrent hemoptysis despite embolization, or when interventional radiology is unavailable^[^22].

Massive hemoptysis carries a poor prognosis if left untreated, with mortality rates reaching as high as 50–60%^[^23]. Timely recognition and management are therefore critical. Although reported cases of Rasmussen’s aneurysm in MDR-TB are rare, the diagnostic approach and management in our case are consistent with previously published literature, reinforcing the role of early CT-PA and prompt endovascular intervention^[^24].

Our case highlights the importance of maintaining a high level of suspicion for Rasmussen’s aneurysm in patients with cavitary lung lesions who present with persistent hemoptysis. It also demonstrates the need for multidisciplinary collaboration among pulmonologists, interventional radiologists, and cardiothoracic surgeons to achieve a positive outcome for high-risk patients. Because this is a single-patient observation, the generalizability of this case may be limited.

## Conclusion

In conclusion, although Rasmussen’s aneurysm is rare, it is an important cause to consider in TB patients with recurrent or unexplained hemoptysis, especially when cavitary disease or MDR-TB is present. Timely diagnosis with CT-PA and early endovascular management can be critical and may be life-saving.

## Data Availability

No data were analyzed or generated in this case study.
